# The effects of the SARS-CoV-2 pandemic on self-reported interoception and mental health

**DOI:** 10.1371/journal.pone.0314272

**Published:** 2025-01-24

**Authors:** Federica Biotti, Melissa Barker, Lara Carr, Hannah Pickard, Rebecca Brewer, Jennifer Murphy

**Affiliations:** 1 Barts and the London Faculty of Medicine and Dentistry, Centre for Psychiatry and Mental Health, Wolfson Institute of Population Health, Queen Mary, University of London, London, United Kingdom; 2 Department of Psychology Royal Holloway, University of London, London, United Kingdom; 3 Centre for Brain and Cognitive Development, Birkbeck, University of London, London, United Kingdom; 4 Department of Psychology, University of Surrey, Guildford, United Kingdom; Anglia Ruskin University, UNITED KINGDOM OF GREAT BRITAIN AND NORTHERN IRELAND

## Abstract

**Background:**

Atypical interoception has been observed across multiple mental health conditions, including anxiety disorders and depression. Evidence suggests that not only pathological anxiety, but also heightened levels of state anxiety and stress are associated with interoceptive functioning. This study aimed to investigate the effects of the recent Coronavirus SARS-CoV-2 pandemic on self-reported interoception and mental health, and their relationship.

**Methods:**

Self-report measures of interoceptive attention and accuracy, anxiety, stress and depression taken during the pandemic (at three time points) were compared to the same measures taken from comparable samples prior to the pandemic. In the sample collected during the pandemic, the relationship between interoceptive and mental health measures and focus on COVID-19-related news and information, propensity to take objective measures of COVID-19 symptoms, and subjective beliefs concerning COVID-19 symptoms was assessed. Finally, a cross-lagged panel model (CLPM) was used to test directional relationships between self-reported interoceptive and mental health measures across three time points.

**Results:**

Higher self-reported anxiety was associated with a) increased self-reported attention to bodily signals, b) increased focus on COVID-19-related news and information, c) propensity to take objective measures of COVID-19 symptoms, and d) reduced self-reported interoceptive accuracy for bodily signals participants believed were associated with COVID-19. The CLPM revealed a mutual and comparable directional effect from T1 to T2 between interoceptive attention and measures of mental health.

**Conclusions:**

Implications of these findings are discussed in the light of existing models and newly proposed accounts of the relationship between interoception and mental health.

## 1. Introduction

On 11^th^ March 2020 the World Health Organization (WHO) declared the outbreak of the novel Coronavirus SARS-CoV-2 a global pandemic. The COVID-19 disease has had a dramatic impact on people’s lives worldwide. The sense of uncertainty and apprehension for one’s own and other people’s health, together with the impact of the pandemic on social relationships and private economies, have led to an increase in self-reported mental health problems, particularly depression and anxiety [1,2], as well as an exacerbation of symptoms in individuals suffering from mental health conditions prior to the pandemic [3]. Notably, evidence suggests that overexposure to COVID-19-related information via social media and news broadcasts was associated with anxiety and stress symptoms both in the general public [4] and in individuals suffering from depression and anxiety [5].

A key aspect of the pandemic’s impact on mental health involves interoception, broadly defined as the processing of one’s own internal bodily states [6]. Indeed, information regarding the wide range of physical internal symptoms associated with COVID-19 was made largely available in an effort to increase public awareness of the disease and foster its containment. The main symptoms associated with COVID-19 were a dry and continuous cough, high body temperature, fatigue, and a temporary loss of taste and smell. In severe cases, patients manifested respiratory symptoms such as shortness of breath and difficulty breathing (Centers for Disease Control and Prevention, 2020). While the risk of contracting COVID-19 has induced anxiety across a large proportion of the population [7], individuals with anxiety sensitivity, who tend to misinterpret changes in bodily signals as potentially dangerous [8], appear to be particularly prone to fear of contracting the disease [7]. This may be due to increased interoceptive attention, as individuals with high anxiety sensitivity exhibit heightened attention to internal bodily states [9]; enhanced attention to bodily sensations known to be associated with COVID-19 may predict an increase in anxiety across the pandemic.

Interoception encompasses both visceral sensations, such as hunger, thirst, and fatigue, as well as signals that derive from external stimulation (e.g. affective touch or taste) but are sent to interoceptive brain areas, such as the anterior insula and the anterior cingulate cortex, via the same afferent pathways used by visceral signals [6,10,11]. As well as involving different bodily systems, interoception is a multifaceted construct which can be broken down into sub-components. One predominant model separates self-report measures (‘interoceptive sensibility’) and behavioural measures of interoceptive accuracy (‘interoceptive sensitivity’), with a final, metacognitive factor reflecting the correspondence between self-reported and objectively assessed interoceptive ability (‘interoceptive awareness’) [12]. More recent proposals, however, highlight a need to not only consider the level of measurement (behavioural versus self-report), but also the facet of interoception assessed by these measures [13]. In these more recent models, a distinction is made between interoceptive accuracy and attention, conceptualised as two distinctive components, with the former reflecting the correspondence between objective performance and the actual interoceptive state (or subjective beliefs regarding this correspondence), and the latter reflecting one’s tendency to take notice of one’s own internal states, again either assessed objectively or through subjective measures [13].

Objective measures of interoceptive accuracy have focused on the use of tasks where participants’ performance is compared with an objective measure of their interoceptive state. In contrast, self-reported measures rely solely on subjective beliefs. While the relationship between self-report measures and interoceptive accuracy has been debated, inconsistent findings may be explained by variation across measurement tools. When subjective beliefs have assessed interoceptive *accuracy*, using either confidence ratings in performance-based tasks [12] or questionnaires that specifically assess interoceptive accuracy such as the Interoceptive Accuracy Scale [14], correlations with behavioural tasks of interoceptive accuracy have been observed. Unsurprisingly, however, measures of self-reported *attention* (e.g., the Body Perception Questionnaire; BPQ; [15] have typically not been associated with performance on behavioural measures of interoceptive accuracy [12,14,16]. Notably, while objective measures of interoceptive attention are lacking, different measures of self-reported attention also appear to correlate with each other [17]. This pattern of relationships within attention and accuracy measures, and lack of relationship across these measurement types, supports the idea that interoceptive accuracy and interoceptive attention are separate factors.

Atypical interoception, defined according to Murphy et al. [18] as “unusually high or low sensitivity, sensibility or awareness” (p. 46), is thought to play a pivotal role in the aetiology and development of a number of mental health conditions, affecting a range of physical and cognitive abilities that are related to specific neurological and psychiatric disorders [11,18–20], such as emotion processing and regulation [21–25], as well as learning and decision-making [26–31]. Indeed, atypical interoception may represent the ‘P factor’ of psychopathology [32] Crucially, a number of signs and symptoms typical of many psychiatric disorders reflect dysfunctional interoceptive states [19]. For example, according to the criteria of the latest Diagnostic and Statistical Manual for Mental Disorders (DSM-5; American Psychiatric Association, 2013), panic disorders are characterised by sensations such as chest pain or discomfort, shortness of breath, nausea, and accelerated heart rate, whereas symptoms of depression include increased or decreased appetite, and fatigue. Higher levels of interoceptive attention, which may alter interpretation of one’s internal signals, are therefore unsurprisingly associated with anxiety disorders such as panic disorder and generalised anxiety disorder [18,33] as well as anxious and depressive symptoms [34], though the relationship between anxiety and objective interoceptive accuracy is mixed [35].

Quantifying self-reported interoceptive ability is particularly relevant in the context of psychopathology. Whilst observed relationships between anxiety and performance on behavioural tasks of cardiac and respiratory interoceptive accuracy have been mixed [36–38]; for a review see [35], anxiety is routinely associated with higher levels of self-reported interoceptive attention and to some extent accuracy [36,39,40]. Such observations have led to the suggestion that anxiety may be related to an atypical pattern of interoceptive ability, characterised by heightened attention relative to objective accuracy [38]. Importantly, subjective beliefs about one’s abilities are likely to guide behaviour in the real world, for example altering one’s attentional focus or motivation and effort when attempting to distinguish interoceptive signals. Self-report measures also have the advantage of assessing interoception across multiple internal signals, while behavioural tasks tend to focus on a single signal channel (e.g. cardiac or respiratory signals).

Associations between self-reported interoception (both in relation to accuracy and attention) and mental health have thus far been investigated exclusively in cross-sectional studies, so the direction of the relationship is thus far unclear. Longitudinal research is therefore required in order to determine the effects of interoception on mental health and vice versa. Research utilising objective measures has, however, investigated causality to some extent. Despite findings suggesting high temporal stability of interoception, at least where objective accuracy is concerned [41,42], there is evidence that not only pathological anxiety, but also heightened levels of situational stress, or state anxiety, can influence objective interoceptive accuracy [43,44]. Stress, in particular, appears to increase interoceptive accuracy on the Heartbeat Counting Task [45–49], though the influence of stress on self-reported interoception is yet to be investigated. Although a transitory stressor does not have a significant long-term effect on the body’s health, exposure to chronic stress or to a major negative event can influence the organism’s neuro-physiological stress response, increasing the risk of psychopathology. Schulz & Vögele [44] have proposed a positive-feedback-loop model to describe the intermediatory role of interoception in the relationship between physiological stress response and the generation of physical symptoms. Crucially, the authors suggest that dysregulated physiological stress response due to chronic stress (or to a major adverse event) may have a direct effect on interoceptive accuracy which, in turn, influences the occurrence of physical symptoms (e.g. palpitation, chest pain). Altered interoceptive accuracy may, therefore, affect interoceptive signals themselves, which may in turn alter interoceptive attention and accuracy further. A major adverse event, and the associated increase in stress, may, therefore, affect interoceptive abilities.

This pre-registered project (https://osf.io/7rncb) aimed to explore whether the COVID-19 pandemic was associated with an increase in individuals’ self-reported interoceptive attention and accuracy by comparing participants’ responses on self-report measures (BPQ and IAS, respectively) pre- (the time prior to the COVID-19 pandemic) and mid-COVID (the time after which the pandemic was declared a national emergency in the U.K., and while strict social distancing rules were in place). This study was structured around five primary aims. First, we investigated whether the COVID-19 pandemic resulted in an increase in self-reported interoceptive attention and accuracy, as evidenced by higher BPQ and IAS scores in a mid-COVID sample relative to an independent pre-COVID sample (Aim 1). Second, we examined whether any differences between pre- and mid-COVID were driven by items assessing bodily signals specifically associated with COVID-19 symptoms (Aim 2). Third, we explored whether any changes in BPQ and IAS scores taken both pre- and mid-COVID were associated with measures of anxiety, depression, and stress, and whether this association was found across the full questionnaires or only for COVID-items (Aim 3). Then, we investigated whether self-reported interoception during the pandemic was associated with a) focus on COVID-related news and information, b) tendency to take objective measures of COVID-19 symptoms, c) correspondence between subjective expectations and the result of objective measures, and d) formal diagnosis of COVID-19 (Aim 4). Finally, in the mid-COVID sample, we explored bi-directional relationships between self-reported interoception and mental health taken at three time points (Aim 5).

## 2. Methods

### 2.1 Samples

#### 2.1.1 Pre-COVID sample

As pre-registered, the pre-COVID sample was obtained from pre-existing data from either published studies [14,50,51] or not yet published research conducted by the authors. The data consisted of anonymised responses from the awareness subscale of the BPQ (long and/or short form) [15], the IAS [14], and the short-form version of the Depression Anxiety Stress Scale (DASS-21; [52]). Data were accessed on 25^th^ June 2020 and were included whenever IP addresses were available, as this prevented the inclusion of data from the same participant. However, the likelihood of overlap of participants across different studies was considered to be minimal, as data were collected by multiple researchers across a range of university locations and participant databases (e.g., Prolific, person databases, Testable Minds). In situations where IP addresses overlapped, data were removed from the database unless they were collected as part of a lab-based assessment, or when there was no overlap of the same questionnaires (e.g., the BPQ was completed in one study and the IAS was completed in another). Where questionnaires were duplicated, the earliest completion was retained. Incomplete data were also excluded from the analyses. Prior to exclusion based on the aforementioned criteria, the total sample included 409 BPQ long awareness scale (hereafter BPQ-long) completions, 1453 BPQ short awareness scale (hereafter BPQ-short) completions, 1193 DASS-21 completions, and 1204 IAS completions. After removal of duplicates, 388 BPQ-long, 1183 BPQ-short, 944 DASS-21, and 1050 IAS completions were retained.

Only participants who completed all the questionnaires were considered for the pre-COVID sample, yielding a sample of 388 individuals. Although not pre-registered, a Malhanobis distance test was performed to identify multivariate outliers, and 2 individuals were removed from the sample due to extreme response patterns in multiple variables. The final pre-COVID sample therefore included 386 people (240 females), 89% of which declared no pre-existing mental health conditions. Age ranged between 18 and 70 years (*M* = 28.02, *SD* = 11.32).

#### 2.1.2 Mid-COVID sample

Data for the mid-COVID sample were collected at three time points. First, we aimed to obtain responses from 388 participants at Time Point 1 (T1) to match the size of the pre-COVID sample. It was anticipated that approximately 30% of participants were likely to agree to take part at the two additional time points as the time commitment required was relatively low. Data collection for T1 started on 5^th^ May 2020 and continued until 2^nd^ June 2020, when the pre-COVID sample size was matched. Data collection for Time Point 2 (T2) occurred 7–9 days after T1 data were collected, and for Time Point 3 (T3) occurred 7–9 days after their T2 data were collected. Participants were recruited via posts on Social Media (e.g., Twitter, Facebook, and Instagram), an advertisement on the research lab’s website (www.Insulab.uk), and through Prolific. Participants were offered the option to take part only once or in all three time points. To incentivise participation, participants could opt to enter a prize draw for vouchers upon completion of each time point of the survey. Participants recruited on Prolific were paid for their participation. Participants needed to be over 18 years old and reside in the United Kingdom at the time of recruitment in order to take part in the study. Participants were informed of the purpose of the study and the procedure prior to take part. An information sheet was provided at the beginning of the study with contact details of the lead researcher and the principal investigator. All participants gave their written consent to participate in the study. The study was approved by the College Research Ethics Committee (REC) at Royal Holloway, University of London (approval code: 2150-2020-05-05-14-42-UFJT034).

A total of 390 participants took part in T1. As above, a Malhanobis distance test was used to identify extreme response patterns on multiple variables and one individual was removed from the analyses. The final mid-COVID T1 sample included 389 participants (243 females), 73% of whom declared no pre-existing mental health conditions. Age ranged between 18 and 77 years (*M* = 34.65, *SD* = 13.24). Of the 389 participants in T1, 119 (79 females) participants completed the study in T2. Participants in the T2 sample were aged between 18 and 73 (*M* = 36.38, *SD* = 13.77), and 69% reported no pre-existing mental health conditions. Finally, the survey in T3 was completed by 86 participants (54 females) aged between 18 and 73 (*M* = 36.72, *SD* = 13.37), and 71% reported no pre-existing mental health conditions.

#### 2.1.3 Sample composition in between-participant analyses

The pre- and mid-COVID samples were not matched according to reported mental health conditions (more mental health conditions were reported in the mid-COVID sample). In order to obtain a closer demographical match, participants who reported a mental health condition were excluded from the between-participants analyses. Given both the timing and context of the mid-COVID data collection, it was deemed unlikely that the between-participant variance in mental health diagnosis was caused by the pandemic itself. Data collection for the mid-COVID group started shortly after the national lockdown began. In such a short period of time and with limited healthcare resources and increased waits for medical appointments [53], a significant increase in formal mental health diagnoses was unlikely. Furthermore, our recruitment strategy may have favoured the participation of people with a mental health diagnosis mid-COVID. The use of the research lab’s social media channels to promote the study may have disproportionally attracted individuals with pre-existing conditions who were more interested in the nature of our research and thus more likely to follow our social media accounts. The pre-COVID sample without individuals reporting mental health conditions was composed of 344 participants (209 females), aged between 18 and 70 (*M* = 27.84, *SD* = 11.40). The mid-COVID sample at TI without individuals with mental health conditions included 271 participants (163 females) aged between 18 and 77 (*M* = 35.00, *SD* = 13.62). The two samples did not differ significantly in gender composition [*X*^*2*^ (1, N = 615) = .02, *p* = .878], but there was a significant difference in age between the two samples [*t* (613) = -7.07, *p* < .001], whereby mean age was higher in the mid-COVID sample (*M* = 35, *SD* = 13.62) than in the pre-COVID sample (*M* = 27.84, *SD* = 11.40). Therefore, age was controlled for in all between-sample analyses, as detailed in the Results section.

#### 2.1.4 Sample composition in mid-COVID within-participant analyses

Only participants who completed the survey at all three time points were included in the within-participant analyses. Participants were required to complete each subsequent survey one week after the previous (a delay in completion of up to two days for T2 and T3 was allowed to minimise data loss). Responses that exceeded this timeframe were excluded from the analyses. This decision was made in retrospect as delays in completion were more prevalent than anticipated at the time the pre-registration was completed.

See Table 1 for a summary of the sample composition and demographic variables of the pre- and mid-COVID samples used in the analyses.

**Table 1:** Samples composition with demographic information.

	PRE-COVID	MID-COVID T1	T1-T2-T3
**Sample size**	344	271	86
**Gender**	209 (60.75%) F 135 (39.25%) M	163 (60.15%) F 108 (39.85%) M	54 (62.79%) F 32 (37.21%) M
**Age mean (SD)**	27.84 (11.40)	35.00 (13.62)	36.72 (13.37)
MHC*	None	None	29% (36% M; 64% F)
**Study design**	Between-participants	Between-participants	Within-participants

*MHC: Mental Health Condition.

### 2.2 Measures

Pre- and mid-COVID changes in self-reported interoceptive were measured using the awareness subscale of the BPQ (Attention) and the IAS (Accuracy), respectively. Two versions of the Attention scale were used in the analyses: the Attention-long consists of 45 items investigating respondents’ awareness of bodily sensations using a 5-point Likert scale (e.g. “During most situations I am aware of muscle tension in my arms and legs”); the Attention-short consists of 12 items taken from the Attention-long. Only 149 participants in the pre-COVID sample completed the Attention-long. The Attention-short was taken from all participants who had completed the Attention-long and from those who only completed the short version in the pre-COVID sample, following the guidelines indicated by Porges [15]. The Accuracy scale includes 21 statements regarding how accurately respondents can perceive specific bodily sensations and responses are based on a 5-point Likert scale (e.g. “I can always accurately perceive when my heart is beating fast”).

In order to compare interoceptive scales across time points, participants completed an additional version of the Attention and Accuracy scales at T1, created for the purpose of the current study (referred to as the Attention-change, Accuracy-change questionnaires). These measures utilised the same items as the standard scales but assessed the extent to which participants’ endorsement of each item had changed in the time since the beginning of lockdown. Participants were instructed to think about how they felt/behaved before the start of lockdown, and how they felt/behave at the time of completing the questionnaire, and responses were made on a 5 point scale indicating the degree of change (in either direction) from before lockdown (S1 File). At T2 and T3, participants received a slightly edited version of the Attention and Accuracy scales with unchanged items and response options, but where they were asked to focus on their feelings/behaviours in the week preceding the completion of the questionnaire.

In order to examine whether Attention and Accuracy items associated with COVID-19 symptoms and sensations (i.e. COVID-items) contributed to global differences of self-reported interoceptive scores between pre- and mid-COVID samples, two new variables were created from each interoceptive scale in addition to the total scores–the Attention-COVID/Accuracy-COVID and the Attention-NonCOVID/Accuracy-NonCOVID scores. The scoring of COVID-items and NonCOVID-items subscales was performed according to the published norms for the main scales (i.e. a high score relates to greater self-reported interoceptive attention/accuracy and vice versa). For ease of comparison among subscales, an average score was calculated by dividing the individual subscales’ total score by the number of items in the subscale. As pre-registered, COVID-items were selected in accordance with the official list of COVID-19 symptoms published by the WHO at the time this study was designed (i.e. May 2020). Specifically, the WHO listed fever, dry cough and tiredness as the most common symptoms. Aches and pains, sore throat, diarrhoea, and headaches were also considered COVID-19 symptoms, although less common. Difficulty breathing and shortness of breath, and chest pain were listed as serious symptoms. At the time this list was published, loss of taste had not been formally recognised by the WHO as one of the COVID-19 symptoms. However, given the substantial anecdotal evidence and media coverage prior to data collection on the possibility that loss of taste could be associated with COVID-19, this symptom was included in the COVID-items list. Currently, the WHO officially recognises the loss of taste as a less common symptom of COVID-19 (https://www.who.int/health-topics/coronavirus#tab=tab_3), justifying the inclusion of this item. Exemplars items of the interoceptive scales (Attention-long, Attention-short, Accuracy, Attention-change, Accuracy-change) and subscales (Attention-COVID, Attention-NonCOVID, Accuracy-COVID, and Accuracy-NonCOVID) can be found in the (S1 File). Personalised COVID- and NonCOVID-item subscales of the Attention scale (p-Attention-COVID, p-Attention-NonCOVID) and Accuracy scale (p-Accuracy-COVID, p-Accuracy-NonCOVID) were also created for the mid-COVID sample, by asking participants to select the symptoms and sensations that they thought were associated with COVID-19 from a list of 43 items (S1 File). This list included all the bodily sensations listed in either the Attention or Accuracy scales. Among the items, 9 were COVID-related symptoms or bodily sensations officially recognised by the WHO (including loss of taste), and 34 were bodily sensations which had been confirmed to be unrelated to COVID-19 symptoms by a medical practitioner, in accordance with the guidelines provided by the WHO.

The DASS-21 was used to collect information regarding mental health. Participants indicated on a 4-point Likert scale the extent to which each statement applied to them over the previous week. The questionnaire includes three subscales (DASS-Anxiety, DASS-Stress, DASS-Depression) with items assessing anxiety, stress and depression, respectively. The DASS-21 was used in its original format in both the pre- and mid-COVID samples, across all three time points.

In the mid-COVID sample, participants’ focus on COVID-related news and information (COVID-Focus) was assessed using five questions investigating the amount of time spent reading, watching, or listening to news and/or COVID-related content (S1 File). Participants responded using a 5-point Likert scale (never, rarely, sometimes, often, very often). At T1, participants responded with reference to the time from the beginning of lockdown until the time of completion. At T2 and T3, participants were prompted to respond with reference to the previous week.

Participants’ tendency to take objective measures of COVID-19 symptoms (COVID-SymptomMeasurement) was assessed by asking participants to indicate how often they had taken objective measures (i.e. taking one’s own body temperature and counting the number of daily coughing episodes), either since the beginning of lockdown (for T1), or during the preceding week (for T2 and T3), using a 5-point Likert scale. In addition, participants were asked to indicate how closely the results of these measurements reflected their expectation (i.e. how accurate their perception of their own symptoms was) on a 3-point Likert scale (COVID-ObjectiveAccuracy) (S1 File).

Finally, participants were asked whether they had ever received a formal diagnosis of COVID-19 (i.e. a positive result on a COVID-19 test). A summary of all variables used in the study can be found in Table 2.

**Table 2:** List of measures.

MEASURE	SAMPLE	MEASURE
**Attention-long**	Pre- and mid-COVID	Interoceptive attention (BPS long form)
**Attention-short**	Pre-and mid-COVID	Interoceptive attention (BPS short form)
**Accuracy**	Pre- and mid-COVID	Interoceptive accuracy (IAS)
**Attention-change**	Mid-COVID	Interoceptive attention change in mid-COVID sample only
**Accuracy-change**	Mid-COVID	Interoceptive accuracy change in mid-COVID sample only
**Attention-COVID**	Pre- and mid-COVID	Pre-defined COVID-related subscale of the BPQ-long
**Attention-NonCOVID**	Pre- and mid-COVID	Pre-defined NonCOVID-related subscale of the BPQ-long
**Accuracy-COVID**	Pre- and mid-COVID	Pre-defined COVID-related subscale of the IAS
**Accuracy-NonCOVID**	Pre- and mid-COVID	Pre-defined NonCOVID-related subscale of the IAS
**DASS-21**	Pre- and mid-COVID	Measure of mental health
**DASS-Anxiety**	Pre- and mid-COVID	Measure of anxiety-related emotions
**DASS-Stress**	Pre- and mid-COVID	Measure of stress-related emotions
**DASS-Depression**	Pre- and mid-COVID	Measure of depression-related emotions
**COVID-Focus**	Mid-COVID	Focus and attention on COVID-19-related news and information
**p-Attention-COVID**	Mid-COVID	Participant-defined COVID-related subscale of the BPQ-long
**p-Attention-NonCOVID**	Mid-COVID	Participant-defined NonCOVID-related subscale of the BPQ-long
**p-Accuracy-COVID**	Mid-COVID	Participant-defined COVID-related subscale of the IAS
**p-Accuracy-NonCOVID**	Mid-COVID	Participant-defined NonCOVID-related subscale of the IAS
**COVID-SymptomMeasurement**	Mid-COVID	Propensity to take objective measures of COVID-19 symptoms
**COVID-ObjectiveAccuracy**	Mid-COVID	Correspondence between expectation and measurement results after objective measure
**COVID-Diagnosis**	Mid-COVID	Presence or absence of previous or current COVID-19 diagnosis

### 2.3 Statistical models

The variables used in the between-participant and within-participant analyses were checked for normality. None of the measured variables were normally distributed as shown by graphical representations of data distribution (i.e. histograms, qq-plots, normal probability plots) and by Kolmogorov-Smirnov tests with *p*_*s*_ < .05. The removal of outliers and mathematical transformations did not yield a normal distribution for most variables, with the only exception of Attention-long and Attention-short, which reached normality after logarithmic transformation. Non-parametric tests were therefore used in the between-participant and within-participant analyses for all variables tested.

To explore directional relationships between self-reported interoception (Attention and Accuracy) and mental health (anxiety, stress, depression DASS-21 subscales) across time, cross-lagged panel models (CLPM) were used. A total of six models were tested, each with three temporal waves (T1, T2, T3) and two levels (interoception, mental health). The models were fitted using the Lavaan package in R [54]. Missing values in each model were accounted for using the method of Full Information Maximum Likelihood (FIML), as it was established that missing values were missing at random (MAR) (i.e. there was no association between the missing variables and other variables in the data). It has been shown that the use of FIML reduces the likelihood of biased parameter estimates and standard errors [55]. In all models, a robust maximum likelihood estimator (MLR) was used to account for data that deviated from normality. The Yuan-Bentler Scaled (χ^2^) test was employed to correct the model test statistic. Goodness of fit was assessed for all models using the comparative fit index (CFI), the root mean square error of approximation (RMSEA), and the standardized root mean square residual (SRMR). CFI values above 0.90 indicate acceptable model fit, whilst values above 0.95 indicate good fit. RMSEA values below 0.06 indicate a good fit between the estimated model and the observed data [56], but note that models with small sample size and low degrees of freedom tend to have artificially high values of RMSEA. SRMR values below 0.08 indicate good fit [57].

**Fig 1:**
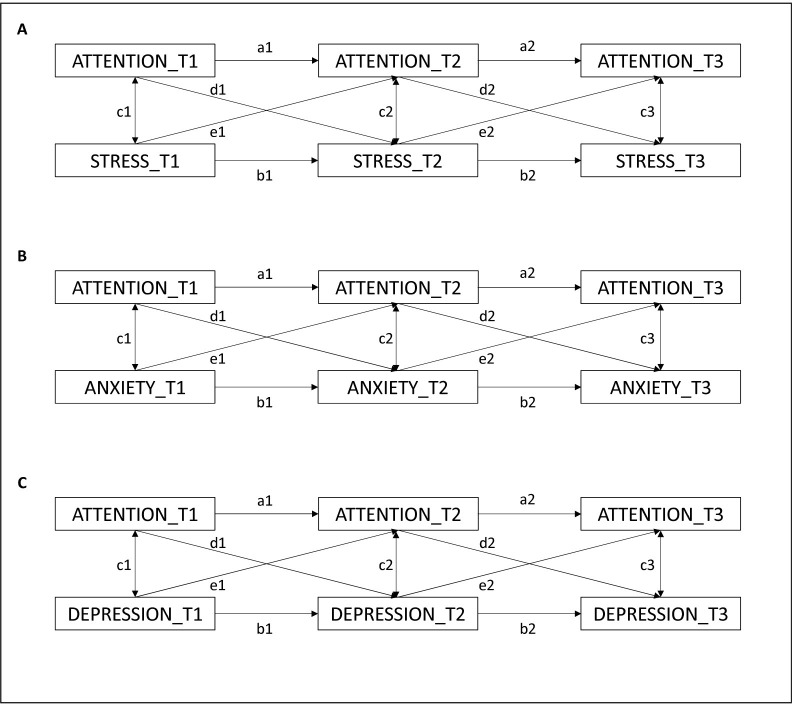
Crossed-lagged panel models estimating the causal relationships between interoceptive attention and stress (A), anxiety (B), and depression (C). The crossed-lagged paths are represented by e and d paths. Autoregressive paths are represented by a and b paths. Covariance paths are represented by c paths.

Autoregressive paths (paths *a* and *b*) estimated the temporal stability of each variable. Covariance paths (paths *c*) estimated the correlation between the two observed variables at each time point. Cross-lagged paths (paths *e* and *d*) estimated the direct influence of one observed variable on the other across time. First, we tested the temporal association between interoceptive attention, and stress (Fig 1A), anxiety (Fig 1B) and depression (Fig 1D) over time.

**Fig 2:**
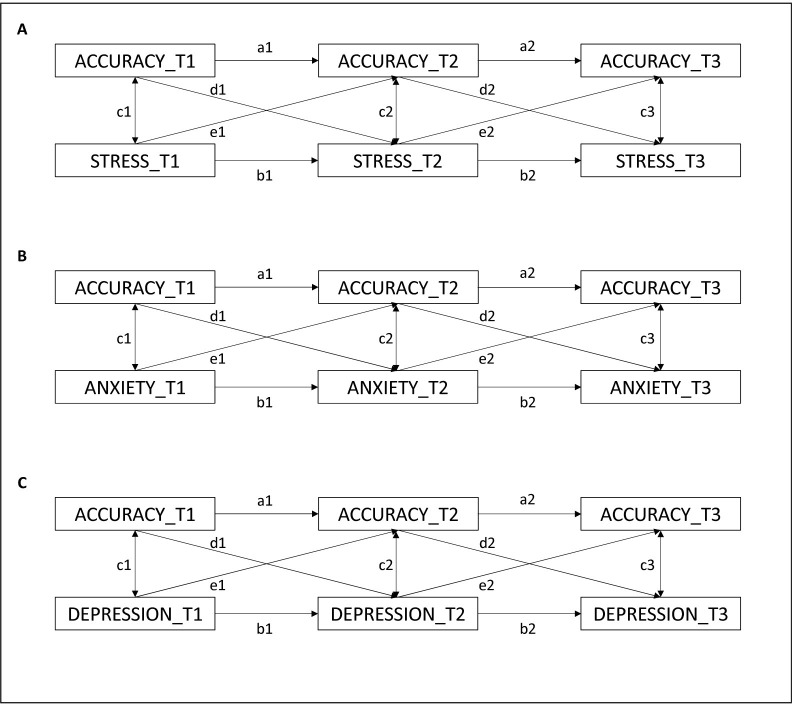
Crossed-lagged panel models estimating the causal relationships between interoceptive accuracy and stress (A), anxiety (B), and depression (C). The crossed-lagged paths are represented by e and d paths. Autoregressive paths are represented by a and b paths. Covariance paths are represented by c paths.

Next, we tested the temporal association between interoceptive accuracy, and stress (Fig 2A), anxiety (Fig 2B) and depression (Fig 2C) over time.

## 3. Results

### 3.1 Between-participant and within-participant results

Between-participant analyses were conducted controlling for age since pre- and mid-COVID groups were not matched. This was a deviation from pre-registration as the age difference was unpredicted.

#### 3.1.1 Research Aim 1: Between-participant differences in self-reported interoception

Analyses of mean differences were conducted using the residuals of the hierarchical multiple regressions used to control for age. A significant difference in Attention-short and Accuracy scores between pre- and mid-COVID samples was found. In the mid-COVID sample (*M* = 4.17, *SD* = .34), participants self-reported higher levels of interoceptive accuracy compared to participants in the pre-COVID sample (*M* = 3.09, *SD* = 1.01) [*U*(N_pre-covid_ = 344, N_mid-covid_ = 270) = 19280.5, *z* = -12.45, *p* < .001]. Conversely, participants in the mid-COVID group (*M* = 2.29, *SD* = .83) self-reported lower levels of interoceptive attention measured with the Attention-short compared to participants in pre-COVID group (*M* = 2.69, *SD* = .93) [*U*(N_pre-covid_ = 344, N_mid-covid_ = 271) = 35616.5, *z* = -5.03, *p* < .001]. The pre- and mid-COVID groups did not differ, however, when interoceptive attention was measured with the long version of the BPQ [*U*(N_pre-covid_ = 143, N_mid-covid_ = 271) = 18185, *z* = -1.03, *p* = .303].

#### 3.1.2 Research Aim 2: The role of COVID-related bodily signals in between-participant interoceptive differences

To examine whether the global differences found in the Attention-short and Accuracy scales were driven by COVID-items, two separate 2 x 2 ANCOVAs were computed with age as a covariate, with Group (pre- vs mid-COVID) as the between-participant factor and Subscale (COVID vs NonCOVID-items) as the within-participant factor. For Attention, a main effect of Subscale was found [*F*(1, 612) = 37.25, *p* < .001, *ηp2* = .057], whereby attention scores were higher for the COVID-items (*M* = 2.76, *SD* = 1.01) than the NonCOVID-items (*M* = 2.39, *SD* = .90) [*Z* = - 15.65, *p* < .001] across groups. In line with the Mann-Whitney U Test above, attention scores were higher in the pre-COVID group than the mid-COVID group [*F*(1, 612) = 27.99, *p* < .001, *ηp2* = .044]. Although there was a significant Subscale x Group interaction [*F*(1, 612) = 5.92, *p* = .015, *ηp2* = .01], post-hoc analyses indicated higher scores in the pre- than mid-COVID sample for both COVID- and NonCOVID-items, and higher scores for COVID-items than NonCOVID-items in both the pre- and mid-COVID samples.

The Accuracy 2 x 2 ANCOVA returned a main effect of Subscale [*F*(1, 611) = 10.04, *p* = .002, *ηp2* = .016], whereby accuracy scores were higher for COVID-items (*M* = 3.63, *SD* = 1.09) than NonCOVID-items (*M* = 3.53, *SD* = .90) [*Z* = - 5.30, *p* < .001]. A main effect of Group was also found [*F*(1, 611) = 220.85, *p* < .001, *ηp2* = .265], whereby accuracy scores were higher in the mid-COVID group than the pre-COVID group. Although a significant Group x Subscale interaction was observed [*F*(1, 611) = 7.10, *p* = .008, *ηp2* = .011], scores were higher in the mid-COVID group than the pre-COVID group for both COVID-items and NonCOVID-items, and higher for COVID-items than NonCOVID-items in both the pre- and mid-COVID groups.

#### 3.1.3 Research Aim 3: Association between self-reported interoception and mental health

To test whether Attention and Accuracy scores taken pre- and mid-COVID were associated with self-reported stress, anxiety and depression, correlational analyses were conducted, both in the combined sample (including both groups), and in the two separate groups. Partial correlations, controlling for age, were conducted in the total sample between Attention-long, Attention-short, Accuracy, and the subscales of the DASS-21 (i.e. Stress, Anxiety, Depression) (Table 3).

**Table 3:** Total sample partial correlations with confidence intervals.

Variable	1	2	3	4	5
**1. Attention-long**					
**2. Attention-short**	.96** [.95 - .97]				
**3. Accuracy**	.04 [-.05 - .13]	.16** [.08 - .24]			
**4. Stress**	.27** [.17 - .35]	.28** [.20 - .35]	.13* [.05 - .21]		
**5. Anxiety**	.30** [.21 - .39]	.36** [.29 - .42]	.16** [.08 - .23]	.66** [.61 - .70]	
**6. Depression**	.15* [.05 - .24]	.16* [.09 - .24]	.17** [.09 - .25]	.68** [.63 - .72]	.54** [.49 - .60]

Values in square brackets indicate the 95% confidence interval for each correlation.

* indicates *p* < .05.

** indicates *p* < .001.

Separate partial correlations were run in the two samples separately, controlling for age (Tables 4 and 5).

**Table 4:** Partial correlations in Pre-COVID sample.

Variable	1	2	3	4	5
**1. Attention-long**					
**2. Attention-short**	.97** [.96 - .98]				
**3. Accuracy**	-.01 [-.18 - .15]	.41** [.32 - .49]			
**4. Stress**	.07 [-.09 - .23]	.24** [.14 - .34]	.24** [.13 - .33]		
**5. Anxiety**	.20* [.03 - .35]	.36** [.27 - .45]	.28** [.18 - .37]	.71** [.65 - .75]	
**6. Depression**	.00 [-.16 - .17]	.17* [.06 - .27]	.24** [.13 - .33]	.68** [.62 - .73]	.57** [.50 - .64]

Values in square brackets indicate the 95% confidence interval for each correlation.

* indicates *p* < .05.

** indicates *p* < .001.

**Table 5:** Partial correlations in Mid-COVID sample.

Variable	1	2	3	4	5
**1. Attention-long**					
**2. Attention-short**	.96** [.96 - .97]				
**3. Accuracy**	.12 [-.00 - .23]	.10 [-.02 - .21]			
**4. Stress**	.37** [.26 - .47]	.33** [.22 - .44]	-.08 [-.20 - .04]		
**5. Anxiety**	.35** [.24 - .45]	.36** [.25 - .46]	-.02 [-.13 - .10]	.59** [.51 - .66]	
**6. Depression**	.23** [.11 - .34]	.19* [.07 - .33]	-.05 [-.16 - .07]	.68** [.61 - .74]	.51** [.41 - .59]

Values in square brackets indicate the 95% confidence interval for each correlation.

* indicates *p* < .05.

** indicates *p* < .001.

A Fisher *R* to *Z* test was used to compare significant correlations in both groups and revealed that the size of correlations did not differ significantly. A Potthoff test was also employed to compare the slopes of the correlations which did not differ in the two groups (S1 Table).

Next, we sought to examine whether the correlations between interoceptive scales and mental health are found globally or only for COVID-items (pre-defined and participant-defined for the mid-COVID sample). In the pre-COVID sample, partial correlations controlling for age revealed that Attention-long-COVID and Attention-long-NonCOVID correlated positively with Anxiety. Both Attention-short (COVID and NonCOVID) and Accuracy (COVID and NonCOVID) correlated positively with all subscales of DASS-21 (Table 6).

**Table 6:** Partial correlations in Pre-COVID sample.

Variable	1	2	3	4	5	6	7	8
**1. Attention-long (COVID)**								
**2. Attention-short (COVID)**	.94**							
**3. Attention-long(NonCOVID)**	.92**	.89**						
**4. Attention-short(NonCOVID)**	.88**	.85**	.95**					
**5. Accuracy-COVID**	-.07	.40**	-.05	.37**				
**6. Accuracy-NonCOVID**	.02	.39**	.01	.39**	.92**			
**7. Stress**	.11	.22**	.05	.24**	.23**	.23**		
**8. Anxiety**	.20*	.29**	.19*	.39**	.28**	.27**	.71**	
**9. Depression**	.01	.14*	-.01	.17**	.26**	.21**	.68**	.57**

* indicates *p* < .05.

** indicates *p* < .001.

In the mid-COVID sample, both Attention-long and Attention-short (COVID- and NonCOVID-items) correlated significantly with Stress, Anxiety, and Depression. However, Accuracy-COVID and Accuracy-NonCOVID did not correlate with any of the DASS-21 subscales (Table 7). In addition, we tested the same correlations using person-defined Attention- and Accuracy-COVID-items. Both p-Attention-COVID and p-Attention-NonCOVID correlated positively with Stress, Anxiety, and Depression. p-Accuracy-COVID and p-Accuracy-NonCOVID both correlated negatively with Stress and Depression, but only p-Accuracy-COVID correlated negatively also with Anxiety (Table 7).

**Table 7:** Partial and bivariate correlations in Mid-COVID sample.

Variable	1	2	3	4	5	6	7	8	9	10	11	12
**1. Attention-long (COVID)**												
**2. Attention-short (COVID)**	.92**											
**3. Attention-long(NonCOVID)**	.90**	.88**										
**4. Attention-short(NonCOVID)**	.86**	.85**	.95**									
**5. Accuracy-COVID**	.20**	.17*	.16*	.15*								
**6. Accuracy-NonCOVID**	.08	.03	.06	.05	.76**							
**7. p-Attention-COVID**	.50**	.45**	.51**	.46**	.07	-.04						
**8. p-Attention-NonCOVID**	.67**	.69**	.76**	.69**	.07	-.02	.66**					
**9. p-Accuracy-COVID**	.16*	.11	.10	.08	.62**	.45**	.03	.06				
**10. p-Accuracy-NonCOVID**	.14*	.10	.08	.06	.53**	.51**	.01	.05	.71**			
**11. Stress**	.36**	.31**	.36**	.33**	-.08	-.07	.24**	.29**	-.17*	-.16*		
**12. Anxiety**	.31**	.30**	.36**	.37**	-.04	.00	.19*	.30**	-.14*	-.08	.59**	
**13. Depression**	.20**	.15*	.23**	.20**	-.09	-.01	.16*	.22**	-.21*	-.22**	.68**	.51**

Bivariate correlations are used with personalised interoceptive scales.

* indicates *p* < .05.

** indicates *p* < .001.

The size of correlations significant in both groups was compared using a Fisher *R* to *Z* test. None of the significant correlations differ between pre- and mid-COVID groups (S2 Table). The slopes of the regression lines did not differ significantly in the two groups, but some of the intercepts were higher for Pre-COVID sample as revealed by a Potthoff test (S2 Table).

In the mid-COVID sample, the extent to which perceived changes in interoceptive attention (Attention-change) and interoceptive accuracy (Accuracy-change) since the start of lockdown were associated with stress, anxiety and depression was investigated. Attention-change scores correlated positively with Stress [*r*_s_ = .33, *p* < .001, *N* = 268], Anxiety [*r*_s_ = .30, *p* < .001, *N* = 268], and Depression [*r*_s_ = .31, *p* < .001, *N* = 268], suggesting greater increases in interoceptive attention since lockdown were associated with poorer mental health. Accuracy-change, on the other hand, did not correlate with any of the DASS-21 subscales. Interestingly, Attention-change and Accuracy-change correlated positively with each other [*r*_s_ = .23, *p* < .001, *N* = 267], suggesting that some participants may have interpreted the two interoceptive scales similarly.

#### 3.1.4 Research Aim 4: The relationship between self-reported interoception and focus on COVID-related information

The extent to which Attention and Accuracy scores (total, COVID-items and NonCOVID-items) correlated with focus on COVID-related news and information (COVID-Focus) was also investigated. COVID-Focus correlated positively with Attention-long-COVID [*r*_*s*_ = .12, *p* = .04, N = 269] but not with Attention-long-NonCOVID [*r*_*s*_ = .08, *p* = .203, N = 269. COVID-Focus did not correlate with either Attention-short (COVID- and NonCOVID-items) nor Accuracy (COVID- and NonCOVID-items). Unplanned analyses (not pre-registered) explored the influence of anxiety on these correlations. Interestingly, when controlling for levels of anxiety (DASS subscale), the partial correlations between Accuracy (total and subscales) and COVID-Focus were significant. COVID-Focus correlated positively with Accuracy total [*r*_*s*_ = .15, *p* = .01, N = 269], Accuracy-COVID [*r*_*s*_ = .14, *p* = .02, N = 269], and Accuracy-NonCOVID [*r*_*s*_ = .15, *p* = .02, N = 269]. The size of the correlation of COVID-Focus with Accuracy-COVID- and Accuracy-NonCOVID did not differ as shown by a Fisher R to Z transformation [(*r*_NonCOVID_ = .15, p = .02, N = 269_;_ *r*_COVID_ = .14, p = .02, N = 269; *r*_NonCOVID_COVID_ = .75, *p* < .001, N = 269), *z* = 0.16, p = .43].

#### 3.1.5 Research Aim 4: The relationship between self-reported interoception, measurements of COVID-19 symptoms, and anxiety

Bivariate correlations between COVID-SymptomMeasurement and interoceptive scales revealed significant positive correlations with the Attention-long (total, COVID- and NonCOVID-items; *p*_*s*_ < .001), and the Attention-short (total, COVID- and NonCOVID-items; *p*_*s*_ < .001). COVID-SymptomMeasurement did not correlate with Accuracy (total, COVID- and NonCOVID-items). Moreover, there was no significant relationship between Accuracy (total, COVID- and NonCOVID-items) and COVID-ObjectiveAccuracy. Interestingly, a significant positive relationship was observed between COVID-ObjectiveAccuracy and p-Accuracy-COVID [*r*_s_ = .18, *p* = .038, N = 128], suggesting that participants who self-report higher accuracy recognising bodily signals they believe are associated with COVID-19, also appear to be more accurate objectively. Finally, unplanned (not pre-registered) analyses investigated the relationship between anxiety and propensity to take objective measures. A positive correlation was found between Anxiety and COVID-SymptomMeasurement [*r*_s_ = .28, *p* < .001, N = 268], suggesting that people reporting higher levels of anxiety were also more likely to take objective measures of COVID-19 symptoms.

As only 7 people in the mid-COVID (2%) reported a formal diagnosis of COVID-19, planned analyses with this variable were not conducted.

### 3.2 Longitudinal results

CLPMs were used to examine the directionality of associations across three time points. The models were tested on a total of 86 participants. Models 1, 2, and 3 assessed the directional influence between self-reported interoceptive Attention, and the DASS subscales Stress, Anxiety and Depression, respectively. Fit indices for the models are reported in Table 8.

**Table 8:** Indices of fit of each model.

Model	χ^2^	Df	*p*	CFI	RMSEA	SRMR
Model 1	14.59	4	.006	.969	.172	.031
Model 2	10.08	4	.039	.983	.133	.020
Model 3	5.69	4	.224	.995	.071	.016
Model 4	22.50	4	< .001	.935	.236	.054
Model 5	17.85	4	.001	.944	.204	.047
Model 6	13.75	4	.008	.971	.171	.042

#### 3.2.1 Research Aim 5: Bi-directional relationship between mental health and self-reported interoceptive attention

Model 1 revealed a bi-directional association between self-reported interoceptive attention and stress at T1 and T2 (paths e1 and d1); Stress at T1 predicted interoceptive attention at T2 (β = .251, *p* = .029, *SE* = .11), and interoceptive attention at T1 predicted Stress at T2 (β = .14, *p* = .02, *SE* = .06). The model did not significantly decrease in fit when the two cross-lagged paths were constrained to be equal (Δχ^2^[df] = 0.67[1], *p* = .41), suggesting both paths had comparable strength.

Autoregressive paths (a1, a2, b1, b2) were all significant, suggesting stability in all interoception and mental health measures over time. Covariance paths between Attention and Stress were significant at T1 and T3 (c1 and c3, respectively), but not at T2 (c2) (Table 9). In Model 2, Anxiety at T1 had a direct effect on self-reported interoceptive attention at T2 (path e1), and interoceptive attention at T1 also had a direct effect on Anxiety at T2 (path d1) (Table 9). Again, no significant decrease in model fit was observed when e1 and d1 were constrained to be equal (Δχ^2^ [df] = 3.24[1], *p* = .07). Interoceptive attention and Anxiety did not influence each other from T2 to T3. Autoregressive paths were all significant (*p* ≦ .001). Interoceptive attention and Anxiety covaried significantly at T1 and T3, but not at T2 (Table 9). In Model 3, self-reported interoceptive attention at T1 predicted Depression at T2 (β = .14, *p* = .024, *SE* = .06). The remaining cross-lagged paths did not reach statistical significance, while the autoregressive paths were all significant (*ps* < .001). Covariance paths were significant at T1 and T3, but not at T2 (Table 9). Models 4, 5, and 6 tested the directional relationships between self-reported interoceptive Accuracy and Stress, Anxiety, Depression, respectively.

**Table 9:** Cross-lagged paths, autoregressive paths, and covariances for each model.

Model	Cross-lagged paths	β (SE)	*p*	Autoregressive paths	β (SE)	*p*	Covariances	β (SE)	*p*
**1**	STRESS_T1 → BPQ_T2* BPQ_T1 → STRESS_T2* STRESS_T2 → BPQ_T3 BPQ_T2 → STRESS_T3	.24 (.11) .16 (.06) .05 (.06) .12 (.07)	.03 .02 .41 .09	BPQ_T1 → BPQ_T2** BPQ_T2 → BPQ_T3** STRESS_T1 → STRESS_T2** STRESS_T2 → STRESS_T3**	.52 (.12) .79 (.09) .70 (.08) .75 (.07)	< .001 < .001 < .001 < .001	BPQ_T1 STRESS_T1* BPQ_T2 STRESS_T2 BPQ_T3 STRESS_T3*	.35 (.06) .14 (.02) .41 (.02)	.001 .191 .003
**2**	ANXIETY_T1 → BPQ_T2* BPQ_T1 → ANXIETY_T2* ANXIETY_T2 → BPQ_T3 BPQ_T2 → ANXIETY_T3	.35 (.15) .19 (.04) -.04 (.12) .09 (.06)	.002 .004 .66 .30	BPQ_T1 → BPQ_T2* BPQ_T2 → BPQ_T3** ANXIETY_T1 → ANXIETY_T2** ANXIETY_T2 → ANXIETY_T3**	.47 (.12) .84 (.10) .72 (.09) .75 (.09)	.001 < .001 < .001 < .001	BPQ_T1 ANXIETY_T1** BPQ_T2 ANXIETY_T2 BPQ_T3 ANXIETY_T3*	.38 (.04) .15 (.01) .39 (.02)	.000 .089 .011
**3**	DEPRESSION_T1 → BPQ_T2 BPQ_T1 → DEPRESSION_T2* DEPRESSION_T2 → BPQ_T3 BPQ_T2 → DEPRESSION_T3	.11 (.07) .14 (.06) -.01 (.07) .00 (.06)	.24 .02 .92 .95	BPQ_T1 → BPQ_T2** BPQ_T2 → BPQ_T3** DEPRESSION_T1 → DEPRESSION_T2** DEPRESSION_T2 → DEPRESSION_T3**	.57 (.11) .82 (.09) .81 (.06) .87 (.05)	< .001 < .001 < .001 < .001	BPQ_T1 DEPRESSION_T1* BPQ_T2 DEPRESSION_T2 BPQ_T3 DEPRESSION_T3*	.35 (.07) .02 (.03) .31 (.02)	.002 .882 .004
**4**	STRESS_T1 → IAS_T2* IAS_T1 → STRESS_T2 STRESS_T2 → IAS_T3 IAS_T2 → STRESS_T3	.32 (.07) .03 (.08) -.02 (.05) .06 (.09)	.002 .66 .81 .31	IAS_T1 → IAS_T2** IAS_T2 → IAS_T3** STRESS_T1 → STRESS_T2** STRESS_T2 → STRESS_T3**	.35 (.08) .76 (.06) .76 (.07) .79 (.06)	< .001 < .001 < .001 < .001	IAS_T1 STRESS_T1* IAS_T2 STRESS_T2 IAS_T3 STRESS_T3	.23 (.04) .07 (.01) -.02 (.01)	.017 .424 .766
**5**	ANXIETY_T1 → IAS_T2* IAS_T1 → ANXIETY_T2 ANXIETY_T2 → IAS_T3 IAS_T2 → ANXIETY_T3	.22 (.09) -.03 (.06) -.11 (.07) -.02 (.08)	.03 .64 .16 .79	IAS_T1 → IAS_T2** IAS_T2 → IAS_T3** ANXIETY_T1 → ANXIETY_T2** ANXIETY_T2 → ANXIETY_T3**	.42 (.08) .78 (.06) .79 (.08) .80 (.07)	< .001 < .001 < .001 < .001	IAS_T1 ANXIETY_T1 IAS_T2 ANXIETY_T2 IAS_T3 ANXIETY_T3	.02 (.04) .20 (.01) -.05 (.01)	.851 .069 .557
**6**	DEPRESSION_T1 → IAS_T2 IAS_T1 → DEPRESSION_T2 DEPRESSION_T2 → IAS_T3 IAS_T2 → DEPRESSION_T3	.19 (.05) -.03 (.08) -.08 (.04) -.06 (.12)	.06 .54 .24 .34	IAS_T1 → IAS_T2** IAS_T2 → IAS_T3** DEPRESSION_T1 → DEPRESSION_T2** DEPRESSION_T2 → DEPRESSION_T3**	.43 (.08) .77 (.05) .86 (.06) .88 (.05)	< .001 < .001 < .001 < .001	IAS_T1 DEPRESSION_T1 IAS_T2 DEPRESSION_T2 IAS_T3 DEPRESSION_T3	-.01 (.05) .01 (.02) -.02 (.01)	.901 .941 .825

* Indicates *p* < .05; ** indicates *p* < .001.

#### 3.2.2 Research Aim 5: Bi-directional relationship between mental health and self-reported interoceptive accuracy

Model 4 revealed that Stress at T1 had a significant effect on self-reported interoceptive accuracy at T2 (β = .32, *p* = .002, *SE* = .07). The other cross-lagged paths did not reach statistical significance. Autoregressive paths were all significant (*ps* < .001). The covariance path at T1 was significant, but covariance paths at T2 and T3 did not reach significance (Table 9). Model 5 showed that Anxiety at T1 had a direct effect on self-reported interoceptive accuracy at T2 (β = .22, *p* = .028, *SE* = .09). The other cross-lagged paths did not reach statistical significance. Autoregressive paths were all significant (*ps* < .001), but none of the covariance paths reached statistical significance (Table 9). In Model 6, none of the cross-lagged paths was significant. The autoregressive paths were all statistically significant (*ps* < .001), but none of the covariance paths reached significance (Table 9).

## 4. Discussion

This study sought to explore the effects of the novel Coronavirus (SARS-CoV-2) pandemic on people’s self-reported interoception and mental health, and their relationship. First, scores on self-report measures of interoceptive accuracy and attention, taken in separate samples before and during the pandemic, were compared. Self-reported interoceptive accuracy was higher during the pandemic (3.1.1), and both self-reported accuracy and attention in the combined groups were higher for bodily sensations associated with COVID-19 symptoms (3.1.2). In the mid-COVID sample, the extent to which self-reported interoceptive accuracy and attention were associated with self-reported mental health, focus on COVID-related information, propensity to take objective measures of one’s own COVID-symptoms, and correspondence between subjective expectations and the result of objective measures, was investigated. Measures of mental health in the combined samples were associated with self-reported interoception. In the mid-COVID group, when only the items related to what participants believed were symptoms and sensations of COVID-19 were considered, results showed that poorer self-reported mental health during the pandemic was associated with reduced self-reported interoceptive accuracy. Moreover, poorer mental health was associated with increased change in interoceptive attention as reported by participants (3.1.3). In turn, increased attention to bodily sensations, as well as higher self-reported anxiety, were both associated with greater propensity to take objective measures of COVID-19 symptoms, such as body temperature and number of coughing episodes. High correspondence between participants’ experience of symptoms and the outcome of objective measure was associated with self-reported interoceptive accuracy for bodily sensations that participants believed were associated with COVID-19 symptoms (3.1.5). Finally, the directional relationships between self-reported measures of interoception and stress, anxiety, and depression were explored. Stress and anxiety reported at the beginning of the study had a directional effect on both self-reported interoceptive attention (3.2.1) and accuracy (3.2.2) measured approximately one week later. In the case of interoceptive attention, this influence appeared to be bi-directional (3.2.1). Depression did not show any relationship with interoceptive accuracy across time, but interoceptive attention reported at the beginning of the study had an effect on how participants felt depressed approximately a week later (3.2.1).

Respondents in the mid-COVID group reported higher levels of accuracy in perceiving their own internal bodily signals compared to those in the pre-COVID group. Increases in interoceptive accuracy during a pandemic is not surprising, particularly in light of evidence showing heightened interoceptive accuracy (both self-reported and objective) in situations of stress [43,44] and anxiety [18,36,39]. Surprisingly, however, in the mid-COVID group, self-reported interoceptive accuracy did not correlate with measures of mental health. A different result was obtained when we used the personalised versions of the accuracy scale, whereby COVID- and NonCOVID-items were determined on the basis of individuals’ selection of signs and symptoms that they believed were associated with COVID-19. Both personalised COVID- and NonCOVID versions of the accuracy scale correlated negatively with stress and depression, but only the personalised COVID version correlated negatively also with anxiety. Whilst a negative relationship between interoceptive accuracy and depression is not surprising [58–62], the negative association between interoceptive accuracy and anxiety conflicts with the positive relationship often observed, at least with objective tests [40,48,63,64]. It is possible that this unexpected relationship is driven by those reporting higher levels of anxiety being more likely to take objective measures of COVID-19 symptoms, such as body temperature and coughing episodes. Taking these measures provides the opportunity for accuracy feedback; if individuals experienced mismatch between objective measures and their expectations (e.g. a normal body temperature detected by a thermometer contrasting with the subjective expectation of having a fever), this may have reduced their perceived interoceptive accuracy. Of course, positive feedback would instead increase perceived accuracy, but if more anxious individuals are taking objective measures with high frequency, the likelihood of having COVID-19 symptoms confirmed on each occasion is low, making negative feedback more likely, in turn lowering interoceptive accuracy scores for COVID-related items. These results suggest that individuals’ beliefs regarding their interoceptive ability are, unsurprisingly, formed on the basis of experience. Receiving objective feedback confirming one’s own sensory expectations promotes beliefs of higher interoceptive accuracy, while experiencing repeated mismatches between perceived sensations and the outcome of objective measures should reduce self-reported interoceptive accuracy. It is possible that beliefs about interoceptive accuracy in turn alter interoceptive attention; perceptions of low accuracy may cause increased attention as a compensatory mechanism. In line with this idea, and with previous findings [39], higher levels of anxiety were associated with increased interoceptive attention. While early models suggested that increased attention to bodily signals would lead to interoceptive oversensitivity [65], more recent evidence suggests that individuals with anxiety have higher levels of interoceptive attention relative to their accuracy [38]. Perhaps individuals higher in anxiety take more frequent objective measures of their internal states, expecting to experience symptoms, providing them with more opportunities to identify low interoceptive accuracy, which in turn increases their interoceptive attention in an attempt to increase accuracy. Notably, while participants were asked whether they took objective measures, they did not clarify whether these measures were taken voluntarily or following a requirement (e.g. within the workplace). This distinction could influence participants’ perception and experience of the process, potentially affecting the interpretation of our findings.

Interestingly, the negative correlation between anxiety and self-reported interoceptive accuracy was only found with the personalised COVID-items, but not with those items in the scale that were associated with bodily sensations that respondents believed were not typical of COVID-19. It is therefore likely that the extent to which one is concerned about specific sensations directly affects one’s perceived accuracy in processing these sensations. An individual with a specific fear of developing COVID-related symptoms, may become oversensitive to sensations associated with these symptoms. The heightened attention to these sensations may lead one to take more frequent objective measurements, which may disprove initial beliefs. The consequent mismatch would decrease the individual’s perceived interoceptive accuracy concerning that specific sensation. Importantly, the same person may show typical levels of interoceptive accuracy (subjective and objective) for bodily sensations that they do not feel anxious about (e.g. hunger and itch, within the context of COVID-related anxiety). Relying on broad questionnaires of interoception, therefore, may not be ideal when studying the relationship with anxiety. Likewise, we may need measures of anxiety that tap onto more specific domains (e.g., physical, social, cognitive).

Self-reported levels of interoceptive attention measured with the short version of the BPQ were lower in the sample collected during the pandemic than in the pre-COVID group. Given the presence of some individual differences between samples (e.g., the groups were not age-matched), results based on the 12-item BPQ should be interpreted with caution. Moreover, below we discuss some limitations of the BPQ as a measure of interoceptive attention.

In the mid-COVID group, self-reported interoceptive attention to pre-defined COVID-related bodily sensations correlated positively with both anxiety and focus on COVID-related news and information. This finding is consistent with a recent study, which found that both BPQ and the number of hours spent viewing COVID-19 media correlated positively with COVID-19 state anxiety [66], and previous findings of a positive relationship between anxiety and interoceptive attention (e.g. [33,48]). These results are also in line with findings of increased self-reported anxiety in the general population following overexposure to news and information about the pandemic via social media [4,67]. These findings support the proposals of interoception as an adaptable and flexible function, contrasting with early notions of interoception as a trait with high temporal stability (e.g., [68]). Specifically, interoceptive attention is likely to be state-depenendant and susceptible to top-down infuence. In some cases, the ability to modulate interoceptive ability in response to environmental changes or cognitive influence can have an adaptive function. For example, individuals who receive a new medical diagnosis might be required to change their interoceptive focus and begin to monitor specific bodily signals in order to manage their condition. Equally, the sudden occurrence of a public health crisis may alter individuals’ attention to specific sensations, serving to minimise illness. Exposure to new information may, therefore, have a causal effect on interoceptive attention. Further research is required, however, to determine the nature of the relationships between news focus, anxiety, and interoceptive attention; it is likely that mutliple bi-directional relationships exist. For example, exposure to particular information may trigger an anxious response which, in turn, prompts attention to relevant bodily sensations, or new information may lead to cosciously increasing one’s interoceptive attention, which then increases anxiety. Likewise, heightened anxiety may cause one to actively search more for information, which in turn increases interocetive attention. Moreover, our results were based on a general measure of anxiety. Future investigations could use different scales tapping onto more specific types/dimensions of anxiety (e.g., health anxiety, anxiety sensitivity).

Indeed, in the current study, mutual and comparable directional effects from baseline (T1; when participants were asked to report on their general levels of attention and accuracy before the pandemic) to T2 (completed a week later, when participants were asked to report on the last week) between interoceptive attention and both anxiety and stress were observed using cross-lagged panel models. Self-reported anxiety and stress levels at baseline predicted ratings of interoceptive attention measured a week later, and self-reported interoceptive attention predicted subsequent self-reports of stress and anxiety. Self-reported stress and anxiety at baseline were also associated with self-reported interoceptive accuracy at T2. These results can be interpreted according to the positive-feedback-loop account [44], whereby physiological stress response due to a major adverse event is thought to have a direct effect on interoception, leading to the occurrence of physical symptoms.

Interestingly, items on both the BPQ and the IAS that were associated with bodily states typical of COVID-19 symptoms received higher ratings than those not associated with COVID-19 across both groups (i.e. even in the group rating their accuracy in perceiving or attention to these signals before they were aware of the COVID-19 pandemic). This may be due to COVID-items referring to interoceptive states that are experienced more frequently. For example, IAS COVID-items include accuracy perceiving signals such as breathing rate, tastes, and temperature, which are encountered on a daily basis. In contrast, NonCOVID-items of the IAS tap into less common (and potentially less easily detectable) bodily sensations such as getting a bruise, having low blood sugar, or being touched affectionately. A similar pattern occurs in the BPQ, whereby COVID-items, tend to refer to frequently encountered internal sensations (e.g., “an urge to cough to clear my throat”, “muscle tension in my back and neck”, “being exhausted”). On the other hand, NonCOVID-items pertain to more infrequent states or sensations (e.g., “a ringing in my ears”, “tremor in my hands”, “my nose itching”). The distinction between more common and uncommon sensations is particularly relevant in the light of interpretation issues with the BPQ–where participants have interpreted the scale as a measure of occurrence of different internal states, differences between COVID- and NonCOVID-items may be particularly large. It is therefore worth noting that some differences between COVID- and non-COVID items in the current study may be explained by frequency of signal occurrence, rather than their relation to COVID-19 *per se*. Of relevance to the broader field of interoception research, this suggests that individual items may, at least in part, drive within- or between-participant differences as recently observed in experience sampling studies [34]. In clinical groups, for example, signal frequency or relevance to the individual or their condition may affect overall questionnaire scores. For example, items referring to breathing frequency or ease would be more relevant to individuals with respiratory conditions, while a far larger set of items is associated with symptoms of conditions such as multiple sclerosis. Individuals’ questionnaire scores may, therefore, be a function of the extent to which each specific signal is experienced or relevant to them.

### 4.1 Limitations

The findings presented in this study come with some methodological and theoretical limitations. First, we found discrepancies in results when different versions of the BPQ were used. A recent study has questioned the validity of the BPQ as a measure of interoceptive attention due to the ambiguity of the word ‘awareness’ used in the questionnaire [69]. This study found that interpretation of this term varied among participants; 30% of respondents believed the BPQ was a measure of accuracy, while 36% believed it was a measure of attention and 31% believed it was a measure of the frequency of occurrence of bodily signals. The fact that the current study identified a small correlation between IAS and BPQ in the combined sample suggests that some participants may have interpreted the BPQ as assessing interoceptive accuracy. While concerns have also been raised about the BPQ potentially being a proxy measure of anxiety, with higher scores on this questionnaire potentially indicating a “maladaptive” type of interoceptive attention [70], the current results are inconsistent with this conceptualisation; levels of anxiety were higher but BPQ scores were lower in the mid-pandemic group than the pre-pandemic group. However, this pattern of results could be due to individual differences in BPQ interpretation as described above, or to increased anxiety found during the pandemic specific to fears surrounding COVID-19, rather than generalised anxiety.

Causal relationships resulting from longitudinal data should also be interpreted with caution. Whilst situational factors have previously been found to account for variance in interoception measured at different temporal occasions, these effects remain relatively small [43]. Moreover, slightly different versions of the interoceptive questionnaires were used at baseline (T1) compared to the other time points. At T2 and T3, participants were asked to retrospectively assess their interoceptive attention and accuracy in the preceding week, but at baseline they rated their general interoceptive ability before the pandemic. Moreover, our results are based on a fairly small sample, and relied on self-reported questionnaires rather than objective measures, questioning the reliability of ratings made retrospectively. Future research is needed to investigate the temporal stability of different facets of interoception, measured both with objective tasks and subjective reports, using more reliable samples and making use of daily ‘in the moment’ reports [34].

Finally, comparisons made between the pre- and mid-COVID groups could be more reliable if the same people were tested. The fact that the two samples were not matched on some demographical variables (e.g., age) is a limitation that needs to be considered when interpreting the current findings, though this was statistically controlled for. Moreover, while the context of a global pandemic is a good opportunity to test interoceptive change in the general population, many individuals experienced changes in their lives as direct or indirect consequences of the pandemic, whose effects could not be measured or controlled.

## 5. Conclusion

To conclude, this study showed a change in self-reported interoceptive attention and accuracy in the general population during the COVID-19 pandemic. This change was particularly evident for bodily sensations thought to be associated with COVID-19 symptoms, and in individuals who reported poorer mental health. Moreover, interoceptive functions and mental health were found to mutually predict each other over time. Our findings contrast with conceptualisations of interoception as a trait with high temporal stability. Instead, we propose that interoception may be an adaptable state-dependent function, influenced by changes in both the internal and external environment, and with a bi-directional relationship with mental health.

## Supporting information

S1 File Scales. (DOCX)

S1 Table Comparisons of significant correlations between pre-COVID-19 and post-COVID-19 groups. Values in the column of Fisher *R* to *Z* test represent *z*-scores. Values in the column of Potthoff test represent *F* statistics. Values in the columns of slope and intercept indicate *t*-test statistics. Asterisks highlight significant tests (* indicates *p* < .05; ** indicates *p* < .001).
(DOCX)

S2 Table Comparisons of significant correlations between pre-COVID-19 and post-COVID-19 groups. Values in the column of Fisher *R* to *Z* test represent *z*-scores. Values in the column of Potthoff test represent *F* statistics. Values in the columns of slope and intercept indicate *t*-test statistics. *P*-values are reported in brackets. Asterisks highlight significant results (* indicates *p* < .05; ** indicates *p* < .001).
(DOCX)
