# Supplementary material for: The effects of the SARS-CoV-2 pandemic on self-reported interoception and mental health
Source: PLoS One. 2025 Jan 24;20(1):e0314272. doi: 10.1371/journal.pone.0314272 (PMC11759990; doi:10.1371/journal.pone.0314272)
Supplement: S1 File — (DOCX) [file pone.0314272.s001.docx]

**S1. Scales**

**S1.1 Body Perception Questionnaire Long Awareness Version (BPQ-long; Porges, 1993)**

Items 3, 6, 11, 15, 16, 19, 21, 22, 31, 34, 35, 38 and 41 were pre-defined as COVID items.

**S1.2 BPQ-change (modified instructions for the BPQ-long)**

Think about how aware you were of your body processes before the start of lockdown (23^rd^ March 2020), and how aware you are now. Select the answer that most accurately describes you.

Rate your change in awareness on each of the characteristics described below using the following five-point scale:

1) Much less aware than before lockdown

2) A bit less aware than before lockdown

3) As aware as before lockdown (no changes)

4) A bit more aware than before lockdown

5) Much more aware than before lockdown

During most situations, since the beginning of lockdown, I am aware of:

**S1.3 Interoceptive Accuracy Scale (IAS; Murphy et al., 2019)**

Items 3, 6, 7, 8, 10, 11, 15, 17 were pre-defined COVID items.

**S1.4 IAS-change (modified version of the IAS)**

Items 3, 6, 7, 8, 10, 11, 15, 17 were pre-defined COVID items.

**S1.5 List of symptoms and sensations^[[1]](#footnote-1)^**

For each of the following symptoms and body sensations, choose an option to indicate whether they are associated or not associated with COVID-19.

We are interested in your knowledge, so refrain from looking for the correct answer elsewhere.

Please select one of the options below for each item:

a) Associated with COVID-19

b) Not associated with COVID-19

1) Hunger

2) Clumsiness

3) Facial twitches

4) Water retention

5) Itchy skin

6) Constipation

**7) Nausea**

**8) Dry cough**

**9) Breathlessness**

10) Urge to urinate

**11) Muscle and joint aches**

**12) Fever (high body temperature)**

13) Hairs on neck standing up

**14) Tiredness/fatigue**

15) Increased belching (burping)

16) Tachycardia (fast heart rate)

17) Increased bruising

18) Difficulty focussing

19) Dry mouth

20) Facial tension

21) Increased sneezing

22) Eye fatigue or pain

23) Bruxism (teeth grinding)

**24) Difficulty breathing**

25) Excessive swallowing

26) Bloated stomach

27) Flatulence (increased wind)

28) Sore throat

29) Swelling of the body or parts of the body

30) Watery/teary eyes

**31) Loss of taste**

32) Change in libido (sex drive)

33) Restlessness

34) Tinnitus (ringing in ears)

35) Hyperhidrosis (excessive sweating)

**36) Diarrhoea**

37) Unusual eye movements

38) Dehydration

39) Dizziness

40) Stomach growling/rumbling

41) Tremor in the hands

42) Tickling or numb skin

43) Touch aversion

**S1.6 COVID-Focus ^[[2]](#footnote-2)^**

Please read each statement and rate how much the statement applied to you since the beginning of the lockdown in the UK (i.e. 23 March 2020) using the following 5 point scale:

a) Never

b) Rarely

c) Sometimes

d) Often

e) Very often

f) Does not apply to me (only for item 5)

Since the beginning of lockdown, how often have you:

1) Checked the news on COVID-19 (for example in newspapers, on social media, on the radio, on TV)?

2) Read/watched/listened to information related to COVID-19?

3) Talked about COVID-19-related topics with other people (for example friends, family, partner, colleagues)?

4) Thought of COVID-19-related events, facts, and information?

5) Published/posted COVID-19-related content on Social Media (for example, Twitter, Facebook, Instagram, YouTube)?

**S1.7 COVID-SymptomMeasurement and COVID-ObjectiveAccuracy ^[[3]](#footnote-3)^**

Indicate how often you have taken the following measures since the beginning of lockdown in UK (i.e. 23^rd^ March 2020) using the following scale:

a) Never

b) Rarely

c) Sometimes

d) Often

e) Very often

1. Body temperature

2. Counting the number of coughing episodes per day

With reference to the question above, how accurately did the results of your measures reflect your expectations? Use the following scale:

a) Not at all accurate

b) Moderately accurate

c) Very accurate

d) Does not apply (I did not take this measure)

1. Body temperature

For example, if you felt as though you had a fever, but when you checked your body temperature, it was lower than you expected, this would be an example of being inaccurate.

2. Counting the number of coughing episodes per day

For example, if you felt as though you had coughed a lot, but when you checked the total number of coughing episodes, it was lower than you expected, this would be an example of being inaccurate.

1. COVID-related symptoms and sensations officially recognised by the WHO (with the addition of loss of taste) are in bold [↑](#footnote-ref-1)
2. At T2 and T3, participants were prompted to respond with reference to the previous week. [↑](#footnote-ref-2)
3. At T2 and T3, participants were prompted to respond with reference to the previous week. [↑](#footnote-ref-3)
