# Supplementary material for: The effects of the SARS-CoV-2 pandemic on self-reported interoception and mental health
Source: PLoS One. 2025 Jan 24;20(1):e0314272. doi: 10.1371/journal.pone.0314272 (PMC11759990; doi:10.1371/journal.pone.0314272)
Supplement: S1 Table — Values in the column of Fisher R to Z test represent z-scores. Values in the column of Potthoff test represent F statistics. Values in the columns of slope and intercept indicate t-test statistics. Asterisks highlight significant tests (* indicates p < .05; ** indicates p < .001). (DOCX) [file pone.0314272.s002.docx]

| Correlation | Fisher (*Z*) | Potthoff (*F*) | Slope (*t*) | Intercept (*t*) |
| --- | --- | --- | --- | --- |
| BPQ-long – DASS-Anxiety | -1.56 | .39 | .62 | 2.11* |
| BPQ-Short – DASS-Stress | -1.2 | .39 | .63 | 1.87 |
| BPQ-Short – DASS-Anxiety | -.04 | .06 | -.25 | 4.12** |
| BPQ-Short – DASS-Depression | -.25 | .07 | .26 | 1.12 |
