# Supplementary material for: The effects of the SARS-CoV-2 pandemic on self-reported interoception and mental health
Source: PLoS One. 2025 Jan 24;20(1):e0314272. doi: 10.1371/journal.pone.0314272 (PMC11759990; doi:10.1371/journal.pone.0314272)
Supplement: S2 Table — Values in the column of Fisher R to Z test represent z-scores. Values in the column of Potthoff test represent F statistics. Values in the columns of slope and intercept indicate t-test statistics. P-values are reported in brackets. Asterisks highlight significant results (* indicates p < .05; ** indicates p < .001). (DOCX) [file pone.0314272.s003.docx]

| Correlation | Fisher (*Z*) | Potthoff (*F*) | Slope (*t*) | Intercept (*t*) |
| --- | --- | --- | --- | --- |
| BPQ-long-COVID – DASS-Anxiety | -1.13 | .20 | .45 | 1.60 |
| BPQ-long-NonCOVID – DASS-Anxiety | -1.77 | .04 | .20 | 2.16* |
| BPQ-short-COVID – DASS-Stress | -1.18 | .28 | .53 | 1.73 |
| BPQ-short-COVID – DASS-Anxiety | -.13 | .10 | -.32 | 3.39** |
| BPQ-short-COVID – DASS-Depression | -.12 | .01 | .08 | 1.04 |
| BPQ-short-NonCOVID – DASS-Stress | -1.19 | .42 | .65 | 1.85 |
| BPQ-short-NonCOVID – DASS-Anxiety | .28 | .04 | -.20 | 4.29** |
| BPQ-short-NonCOVID – DASS-Depression | -.38 | .12 | .35 | 1.11 |
